# Relationships between a common Caribbean corallivorous snail and protected area status, coral cover, and predator abundance

**DOI:** 10.1038/s41598-020-73568-1

**Published:** 2020-10-05

**Authors:** Elizabeth C. Shaver, Julianna J. Renzi, Maite G. Bucher, Brian R. Silliman

**Affiliations:** 1 The Nature Conservancy, Arlington, VA USA; 2 Division of Marine Science and Conservation, Nicholas School of the Environment, Duke University, Beaufort, NC USA; 3 Department of Marine Sciences, College of Arts and Sciences, University of North Carolina at Chapel Hill, Chapel Hill, NC USA

**Keywords:** Food webs, Marine biology, Conservation biology

## Abstract

As coral populations decline across the Caribbean, it is becoming increasingly important to understand the forces that inhibit coral survivorship and recovery. Predation by corallivores, such as the short coral snail *Coralliophila abbreviata*, are one such threat to coral health and recovery worldwide, but current understanding of the factors controlling corallivore populations, and therefore predation pressure on corals, remains limited. To examine the extent to which bottom-up forces (i.e., coral prey), top-down forces (i.e., predators), and marine protection relate to *C. abbreviata* distributions, we surveyed *C. abbreviata* abundance, percent coral cover, and the abundance of potential snail predators across six protected and six unprotected reefs in the Florida Keys. We found that *C. abbreviata* abundance was lower in protected areas where predator assemblages were also more diverse, and that across all sites snail abundance generally increased with coral cover. *C. abbreviata* abundance had strong, negative relationships with two gastropod predators—the Caribbean spiny lobster (*Panulirus argus*) and the grunt black margate (*Anisotremus surinamensis*), which may be exerting top-down pressure on *C. abbreviata* populations. Further, we found the size of *C. abbreviata* was also related to reef protection status, with larger *C. abbreviata* on average in protected areas, suggesting that gape-limited predators such as *P. argus* and *A. surinamensis* may alter size distributions by targeting small snails. Combined, these results provide preliminary evidence that marine protection in the Florida Keys may preserve critical trophic interactions that indirectly promote coral success via control of local populations of the common corallivorous snail *C. abbreviata.*

## Introduction

Climate change and human exploitation are driving dramatic declines in reef-building hard corals across the globe^1–3^. In an effort to mitigate losses in coral cover and inform conservation efforts, the conservation community is seeking to identify ecological processes that promote coral survivorship. Although environmental factors (e.g., temperature, pH) are often globally dictated and difficult to control, top-down factors (e.g., predation, herbivory) can be manipulated on the scale of a marine protected area (MPA) or restoration project. For instance, by protecting herbivores, MPAs can indirectly facilitate coral health by reducing the abundance of macroalgal species that compete with corals^4–7^. However, top-down interactions that could promote coral reef recovery, such as protection of predators that feed on and control populations of coral-eating animals (i.e., corallivores), are less understood. While recent evidence shows that corallivores can reduce coral fitness^8^ and resilience to warming events^9–11^, we still have little understanding about the factors that influence local densities of coral predators.

Much like macroalgae, corallivores naturally occur in healthy coral reef ecosystems but can devastate coral populations when overabundant. Population outbreaks of crown-of-thorns starfish (COTS) in the Indo-Pacific, for instance, have led to declines in coral cover from > 40% cover to < 5% cover in some areas^2,12^. High densities of the corallivorous snails *Drupella* spp. and *Coralliophila* spp. have significantly impacted corals in the Indo-Pacific, Western Indian Ocean, and Asia^13–15^. Similarly, in the Caribbean, predation by the short coral snail *Coralliophila abbreviata* has been responsible for inhibiting the recovery of coral populations^32,34^, particularly after disturbances such as severe weather events^16,17^. While environmental factors have been cited as mechanisms that increase some corallivore populations (e.g., nutrients in run-off leading to increased larval survivorship of COTS^18^), overfishing of higher trophic-level predators that prey on corallivores may also contribute to corallivore outbreaks^19–21^.

The mass mortality of the once-dominant reef building corals *Acropora cervicornis* and *A. palmata* in the 1980s was responsible for significant reductions in coral cover across the Caribbean region^22–24^. Despite region-wide coral reef protection and restoration efforts, predation by corallivores like *C. abbreviata* can impede the recovery of critical coral populations^17,25^. For instance, high densities of this corallivore may harm corals through sublethal predation effects, direct tissue loss, and disease vectoring^26–31^, which have important implications for both reef establishment and resilience. For example, after Hurricane Allen in Jamaica, *C. abbreviata* aggregated on and devastated remaining colonies of *A. cervicornis*, hindering the recovery of these corals after the storm^16^. Similarly, Caribbean brain corals preyed upon by *C. abbreviata* snails were less resilient and more severely damaged following a warming event compared to colonies in which snails were manually removed prior to bleaching^9^. Management agencies such as the US National Oceanic and Atmospheric Administration have included manual removals of *C. abbreviata* as part of their *Acropora* spp. recovery and management plans, reinforcing the important role these corallivores play in reef dynamics^32^. However, the density of these corallivorous snails across sites and the possible factors regulating their abundance has not been described, particularly in the context of top-down and bottom-up forces.

Several studies have examined the role of fishing pressure on corallivorous invertebrates, often finding lower corallivore abundance in reefs protected from fishing^15,19,21,33^. However, this research has been focused on Pacific reef systems, with no study to date examining the role of site protection on densities of *C. abbreviata* in the Caribbean. Further, little research has been undertaken to assess the relative importance of both top-down (e.g., predation) and bottom-up (e.g., coral prey) factors on corallivore abundance. Thus, despite their global importance and threat to degraded coral populations, we do not fully understand the mechanisms governing local corallivore abundances. For *C. abbreviata*, there is also limited knowledge of the identity of their predators*.* Besides the deltoid rock snail *Thais deltoidea,* which was recently been identified preying on *C. abbreviata*^34^, predatory species have merely been suspected but not been experimentally assessed^30,35^.

To begin to understand how local abundances of Caribbean corallivorous snails are influenced by biological factors, we surveyed *C. abbreviata*, hard corals, and 13 potential *C. abbreviata* predators in 6 protected (fishing prohibited) and 6 unprotected (fishing allowed) reefs across the Florida Keys, USA. We then examined how bottom-up factors, top-down factors, and the effect of site protection status influenced local *C. abbreviata* abundance. To identify potential candidate predators of *C. abbreviata*, we also examined correlations between the abundances of *C. abbreviata* and individual gastropod-predators.

## Methods

### Study design

**Figure 1:**
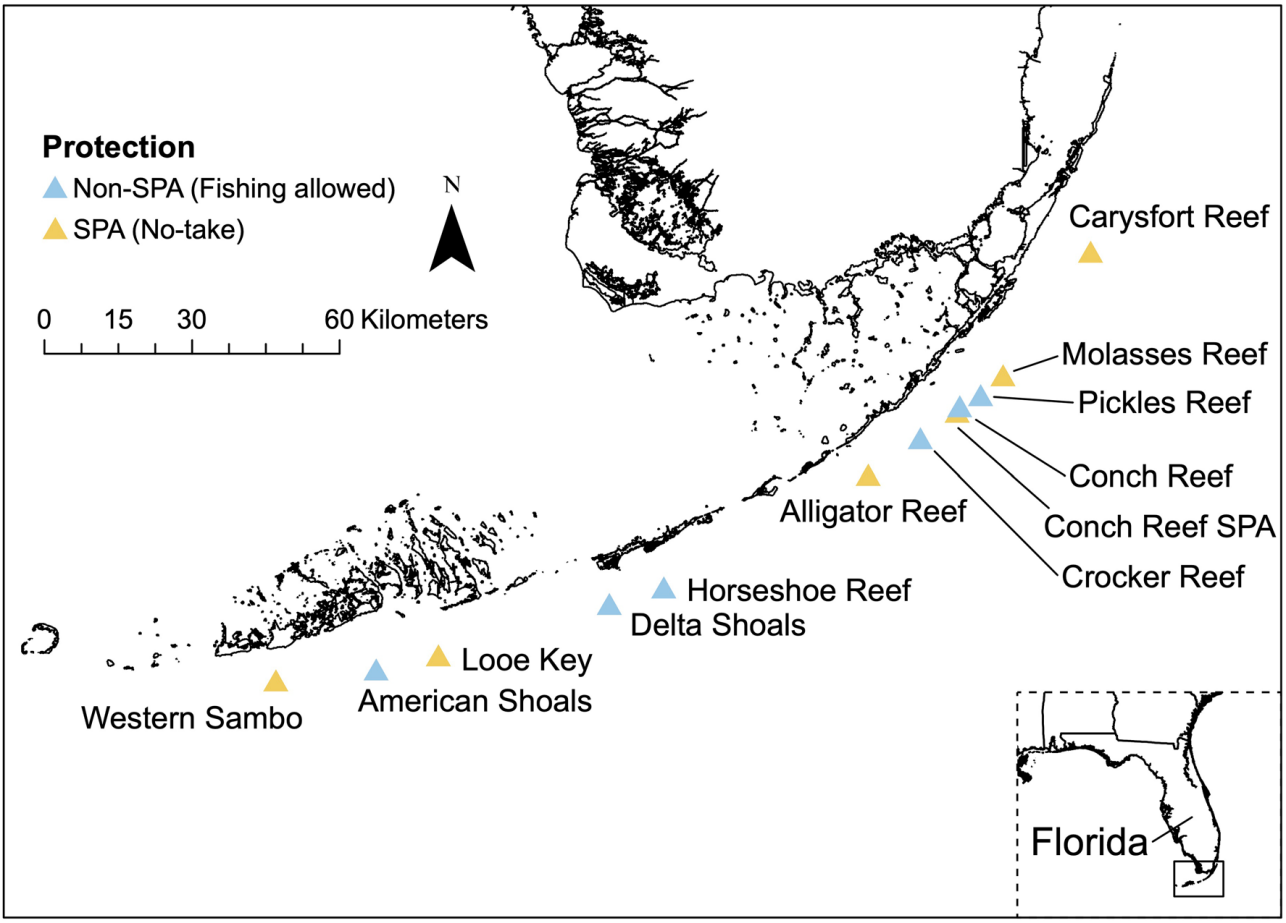
Map of the coral reef sites where surveys were conducted throughout the Florida Keys. The map was generated using ArcGis Pro 2.5.0 (Copyright Esri Inc.) with the shoreline layer from the NOAA National Ocean Service (available on ArcPortal).

To examine how bottom-up and top-down factors are related to local abundances of *Coralliophila abbreviata*, we surveyed 12 coral reefs that spanned the reef tract of the Florida Keys National Marine Sanctuary, USA (Fig. 1). Although all reefs within the sanctuary are protected by some level of regulation and management, our survey sites included 6 reefs specifically designated as no-take zones called Sanctuary Preservation Areas (hereafter called “SPAs” or “protected” sites; Fig. 1) where fishing, harvesting, or possessing any marine life is prohibited^36^. Fishing is allowed within the other 6 sites surveyed (hereafter called “non-SPAs” or “unprotected” sites), which previous studies have found support lower fish abundances than protected sites^37,38^. All sites were part of the outer barrier reef system and surveys were conducted on the main portion of the shallow reef structure (< 30 m in depth), including reef flats, edges, and spur and groove formations.

All surveys were conducted between June and July 2015. Surveys were performed in accordance with relevant guidelines and regulations. The permit FKNMS-2015-064 was obtained from Florida Keys National Marine Sanctuary and the special activity license SAL-14-1580-SR was obtained from the Florida Fish and Wildlife Conservation Commission to conduct this research.

### Corallivore surveys

**Figure 2:**
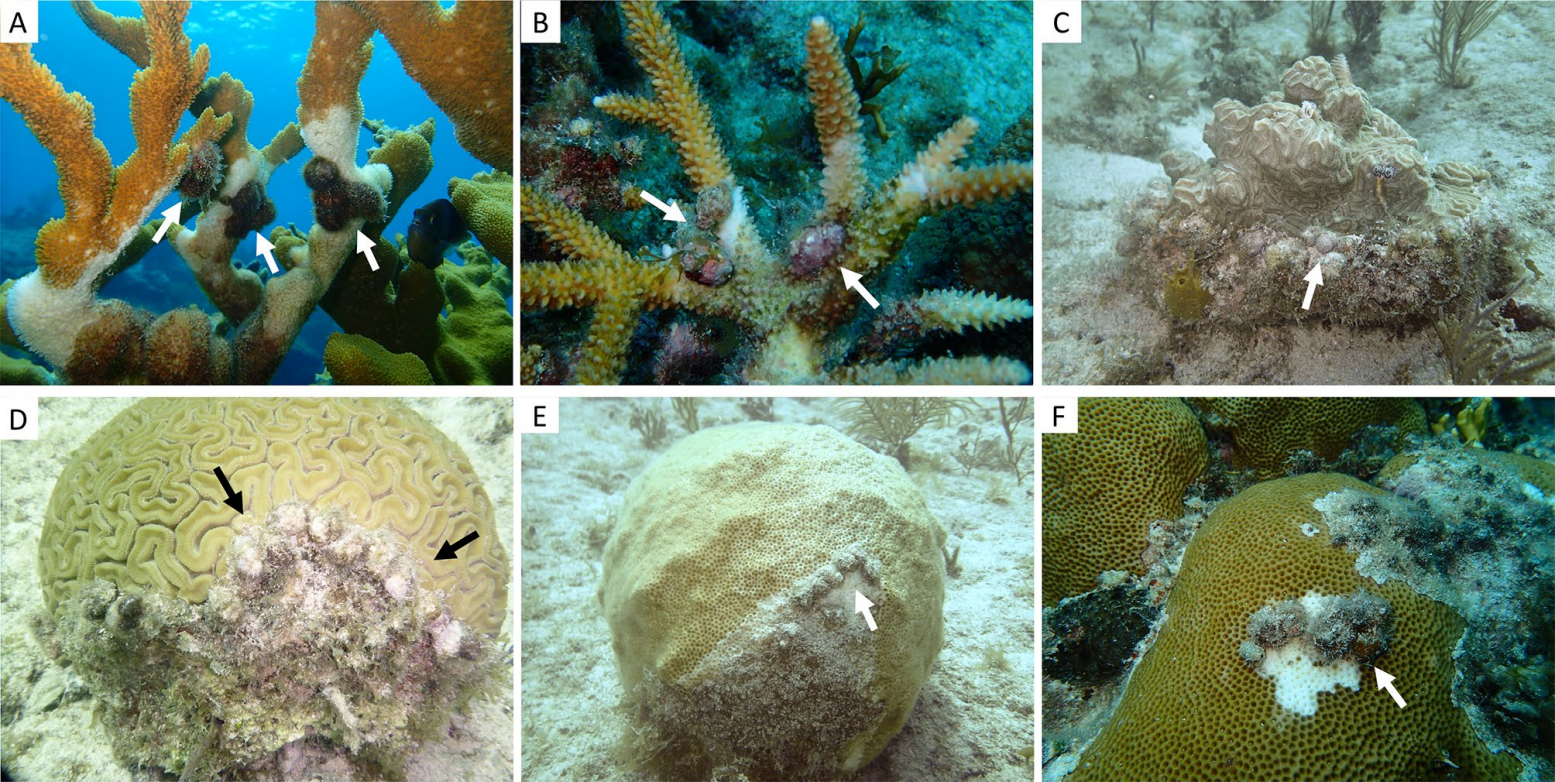
*Coralliophila abbreviata* found consuming different coral prey species, including (**a**) *Acropora palmata*, (**b**) *Acropora cervicornis*, (**c**) *Pseudodiploria clivosa*, (**d**) *Diploria labyrinthiformis*, (**e**) *Solenastrea bournoni*, and (**f**) *Siderastrea siderea*. Arrows indicate the location of *C. abbreviata.* Photo credits: Elizabeth Shaver.

Local abundances of *C. abbreviata* were assessed using visual SCUBA surveys. At each site, two divers examined all living coral colonies located within 12–15, 20 × 2 m belt transects spaced approximately 15 m apart for the presence and abundance of *C. abbreviata*. The exact number of transects surveyed varied based on the size of the site and logistical constraints. On every coral present within each transect, divers visually examined and inspected the margins between living coral tissue and dead coral skeleton or the seafloor for the presence of snails, as *C. abbreviata* often inhabit these margins and consume a variety of coral prey species^9,29,39^ (Fig. 2). Inspection included lightly touching the margins of dead coral skeleton or seafloor to feel for snails, being careful not to touch living coral tissue. The coral species on which *C. abbreviata* were found was also recorded. All *C. abbreviata* were counted, removed, and transported to land for biometric measurements. Counts of snails were pooled for each site and standardized for the number of transects surveyed.

On land, we measured *C. abbreviata* shell length, shell thickness, and live snail biomass (i.e., including shell), after which snails were released. Shell length was measured as the distance from the apex of the shell to the bottom of the operculum, while shell thickness was measured as the thickness of the operculum’s outer lip 5 mm into the aperture. Both metrics were measured using digital calipers. Biomass of live snails was measured using a digital scale.

### Bottom-up factors: hard corals

Total percent coral cover at each site was determined by collecting and analyzing benthic cover photos. To do this, we photographed 40, 0.5-m^2^ plots located within 13–15, 20 × 1-m belt transects spaced at least 5 m apart. The number of transects per site varied based on the size of the reef. All photos were taken at the same height above the seafloor using a PVC apparatus attached to an underwater camera that pointed at the seafloor. All photographs were analyzed for percent cover of all coral species using Microsoft Powerpoint and Excel 2016 software with 25 random points on each slide to identify coral species and estimate cover. Each point was coded to the species level for corals. If points fell on top of objects that were above the seafloor (e.g., queen conch shells), points were moved to the closest area showing benthic cover.

### Top-down factors: predators

To examine how much of the variation in *C. abbreviata* abundance could be explained by predator abundance, we conducted surveys of known gastropod-consuming fishes and other suspected predators of *C. abbreviata*, as actual data on of *C. abbreviata* predators are limited^31,40^. We used existing scientific literature and books to identify potential predators if they met one of three requirements: (1) previous research suggested they may be *C. abbreviata* predators, (2) previous research observed the species preying on *Coralliophila* spp., or (3) the species is within a taxa known to specialize in feeding on marine gastropods. The following species were thus included in surveys as potential *C. abbreviata* predators: Caribbean spiny lobster (*Panulirus argus*^30,35,41^), Family Labridae: slippery dick (*Halichoeres bivittatus*^42^), puddingwife (*Halichoeres radiatus*^42^), hogfish (*Lachnolaimus maximus*^42^), Spanish hogfish (*Bodianus rufus*^42^); Family Haemulidae: white grunt (*Haemulon plumineri*^42^), bluestriped grunt (*Haemulon sciurus*^42^), Caesar grunt (*Haemulon carbonarium*^42^), Spanish grunt (*Haemulon macrostomum*^42^), sailor’s choice (*Haemulon parra*^42^), porkfish (*Anisotremus virginicus*^42^), black margate (*Anisotremus surinamensis*^42^); and Family Diodontidae: porcupinefish (*Diodon hystrix*^30,42^). Queen triggerfish (Family Balistidae, *Balistes vetula*^42^) and balloonfish (Family Diodontidae, *Diodon holocanthus*^42^) were also surveyed but were not observed in any site and were therefore excluded from further analyses. Due to logistical constraints, we were restricted from surveying some cryptic species that have also been cited in the literature as potential *C. abbreviata* predators, including octopuses^30^ and the snapping shrimp, *Synalpheus fritzmuelleri*^43^.

Fish surveys were conducted using the Stationary Point Count method^44^, where two divers are stationary along a 30-m transect at 7.5 and 22.5-m and each surveys a 15-m diameter circle over a given time interval. At each site, we conducted 10 fish surveys spaced at least 20 m apart, allowing for a 5-min acclimation period after which all species identified as potential predators of *C. abbreviata* were counted for 5 min. Surveys of the spiny lobster *P. argus* were conducted using timed-diver survey methods, where 1–2 SCUBA divers systematically swam over the reef site (where fish and snail surveys were conducted) and all *P. argus* observed during a 1-hour total period were recorded. When there were multiple divers, the time was split accordingly. These surveys took place before the 2-day recreational “mini-season” for spiny lobster from 29–30 July 2015 to avoid artificial differences in lobster abundance within and outside of SPAs. Underwater visibility was consistently high during this time period, allowing divers to conduct a clear visual census in each survey area. All surveys were conducted during the day between 1000 and 1600 h. No nighttime surveys were conducted due to lack of resources.

### Statistical Analysis

*Corallivore abundance as a function of protection, coral cover, and predators—*To examine the role of bottom-up forces (i.e. available coral food resources), top-down forces (i.e. presence of predators), and site protection status on local *C. abbreviata* abundance, we created a generalized linear model with a negative binomial distribution to describe snail abundance with survey size as an offset using the R packages MASS^45^ and foreign^46^. We used relative percent coral cover as a proxy for bottom-up forces, total abundance of all potential snail predators surveyed per site as a proxy for top-down forces, and SPA-designation to represent protection as explanatory variables in our model. Total cover of all coral species was included given that this survey and previous surveys in the same sites^9^ have observed *C. abbreviata* preying on a large variety of coral species, including nearly all coral species that were surveyed. A negative binomial distribution was used due to overdispersion with Poisson models. We used protection, coral cover, and predator abundance as fixed effects in the initial model and then created additional plausible models by removing parameters and adding potential two-way interactions, ultimately selecting the most parsimonious model with the lowest AIC value. All analyses were conducted in R version 4.0.0^47^ with the tidyverse suite of packages^48^.

*Corallivore size as a function of protection, coral cover, and predator abundance –* All biometric measurements taken of *C. abbreviata* (e.g., shell length, thickness, and live snail biomass) covaried to some degree. We therefore conducted further analyses using only measurements of snail shell length. This is consistent with previous research examining *C. abbreviata* life history using measurements of shell length for size^40^. Shell length is also the measurement most likely to affect gape-limited gastropod predators. We examined the role of site protection, coral cover, and predator abundance on *C. abbreviata* size distributions using a general linear model with a Gaussian distribution and identity link to describe snail shell length. All variables were included as fixed effects in the initial shell length model. We then created alternate plausible models by removing parameters and adding potential two-way interactions, ultimately selecting the most parsimonious model with the lowest AIC value.

*Predator abundance and species richness between protected and unprotected reefs* -We additionally examined whether there were alternative characteristics of the predator community that might be driving snail distributions in and out of protected areas. To test whether the abundance and species richness of potential *C. abbreviata* predators varied due to site protection status, we performed Welch two-sample t-tests with Shapiro tests to check for normality and F tests to check for equal variances. Predator abundance was log-transformed to better meet assumptions of normality.

*Corallivore abundance as a function of specific potential snail predators*—The predators surveyed were a group of species believed to be most likely to prey on *C. abbreviata*, though the identity of actual predators remained unknown. Given that the global models above assessed total predator abundance in each site and it is unlikely that all species surveyed are predators of *C. abbreviata*, and that a major goal of this study was to identify *C. abbreviata* predators, we examined whether *C. abbreviata* abundance was related to any of the potential predator species surveyed. To examine these relationships, we created generalized linear models with negative binomial distributions to describe snail abundance as a function of each predator species’ abundance with survey size (for snail surveys, which varied) as an offset. A Bonferroni correction was applied to adjust for multiple comparisons of individual predator species (n = 13), with a new alpha value of *P* < 0.0038.

## Results

### Corallivore abundance is related to coral cover and protection status

**Figure 3:**
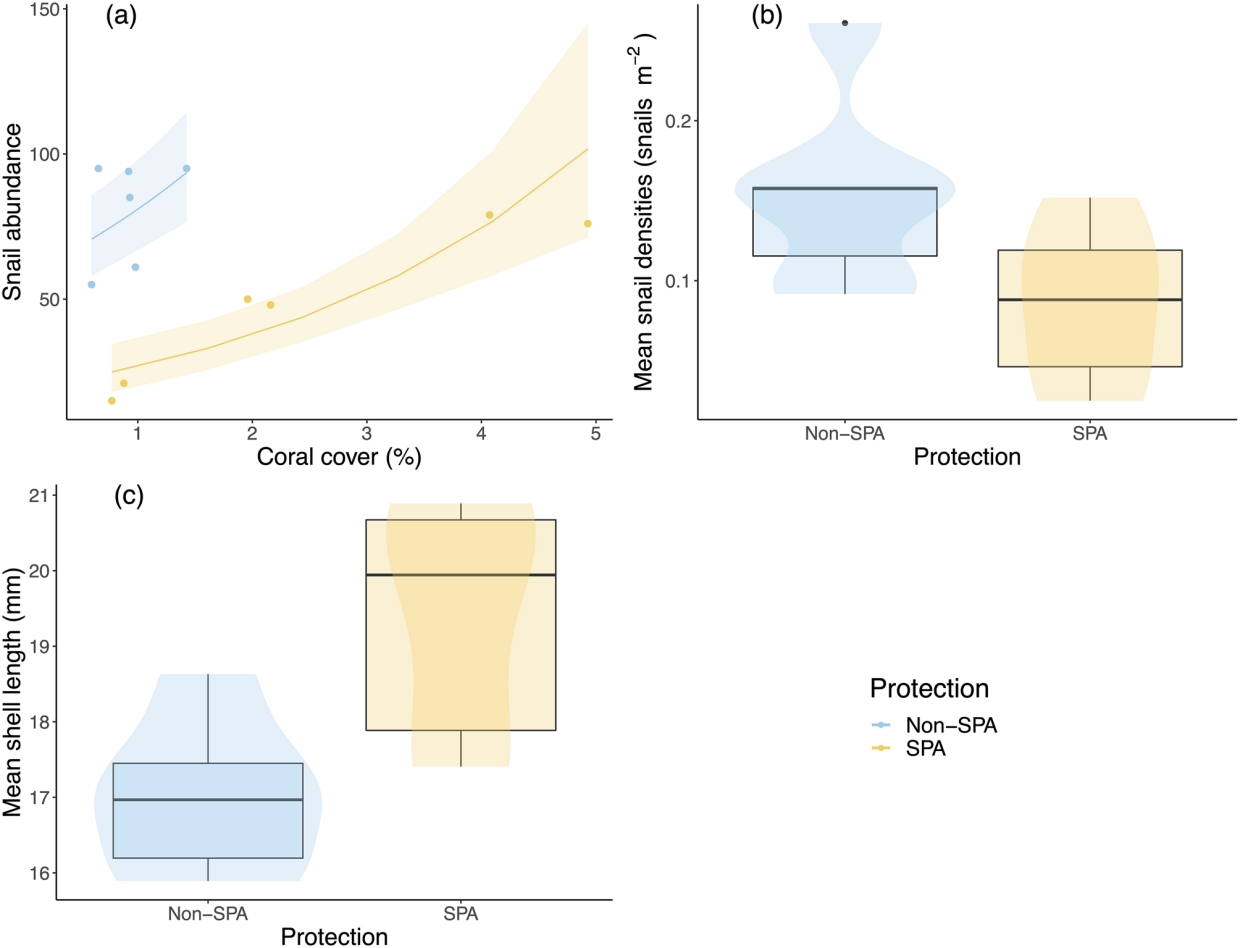
Differences in the number and length of *C. abbreviata* based on site protection status (SPA versus non-SPA) and coral cover. Panel (**a**) shows the relationship between coral cover and snail abundance with 95% confidence intervals. Panels (**b**) and (**c**) are boxplots overlain with a violin plots to show mean snail density (per m^2^) and length inside versus outside of SPAs.

Across all 12 survey sites, the mean density of *Coralliophila abbreviata* snails was 0.120 ± 0.06 per m^2^ (data are reported as mean ± SD for all results; see Supplementary Table S1 online for density per site). Analyses showed that *C. abbreviata* abundance was strongly related to both relative percent coral cover (z_9_ = 5.12, *P* < 0.0001; Fig. 3A) and site protection status (z_9_ = -5.90, *P* < 0.0001; Fig. 3B), while predator abundance appeared less important and was dropped from the model during the selection process. Snail abundance was positively correlated with coral cover but negatively correlated with site protection status. While total percent coral cover was low across all survey sites (1.7 ± 1.4% coral cover), it generally appeared higher in protected areas (SPAs: 2.5 ± 1.7% cover; non-SPAs: 0.9 ± 0.3% cover). Total *C. abbreviata* density and total percent coral cover values for individual sites are given in Supplementary Table S1 online.

Overall, we found that snails were more abundant in unprotected reefs (non-SPA: 0.155 ± 0.060 snails m^−2^; SPA: 0.086 ± 0.050 snails m^−2^). Specifically, a SPA designation was associated with a 3-fold decrease in snail abundance per m^2^ when coral cover was held constant (Fig. 3B).

### Corallivore size is also related to protection status

Snails averaged 17.87 ± 4.91 mm in mean shell length across the 12 survey sites. The most parsimonious global model described snail length as a function of site protection status (t_9_ = 2.68, *P* = 0.025) and predator abundance, although predator abundance was not significant (t_9_ = − 1.44, *P* = 0.184). Although *C. abbreviata* abundances were lower in protected areas, *C. abbreviata* individuals were larger (SPA: 19.28 ± 5.45 mm mean shell length; non-SPA: 16.88 ± 4.24 mm mean shell length), with a SPA designation increasing mean snail length by 2.08 ± 2.69 mm (Fig. 3C).

### Protected areas have increased predator richness but not increased abundance

**Figure 4:**
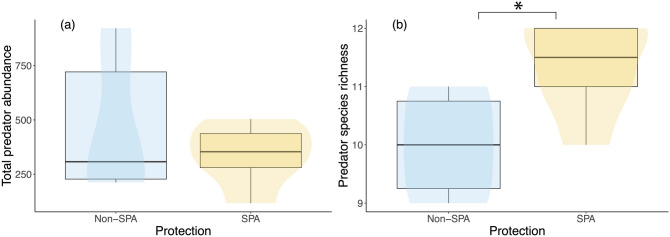
Boxplots overlain with violin plots showing predator abundance and richness inside and outside of protected areas. Asterisk symbolizes statistical significance (*P* = 0.01).

Given that the global models selected protected areas, but not total predator abundance, as important for explaining *C. abbreviata* abundance, we examined whether there were alternative characteristics of the predator community that might be affecting snail distributions in and out of protected areas. We found that the total abundance of all potential predators surveyed was not different due to site protection status (t_9.45_ = 0.62, *P* = 0.550; Fig. 4A). However, predator species richness was significantly higher in protected areas (t_10_ = − 2.70, *P* = 0.022; Fig. 4B), suggesting that certain species may only be present in protected areas. Specifically, two species of grunts (Family Haemulidae), including sailor’s choice (*Haemulon parra*) and black margate (*Anisotremus surinamensis*), were not present in unprotected areas at all, while others were less commonly found in unprotected sites.

### Potential predators of *C. abbreviata*

**Figure 5:**
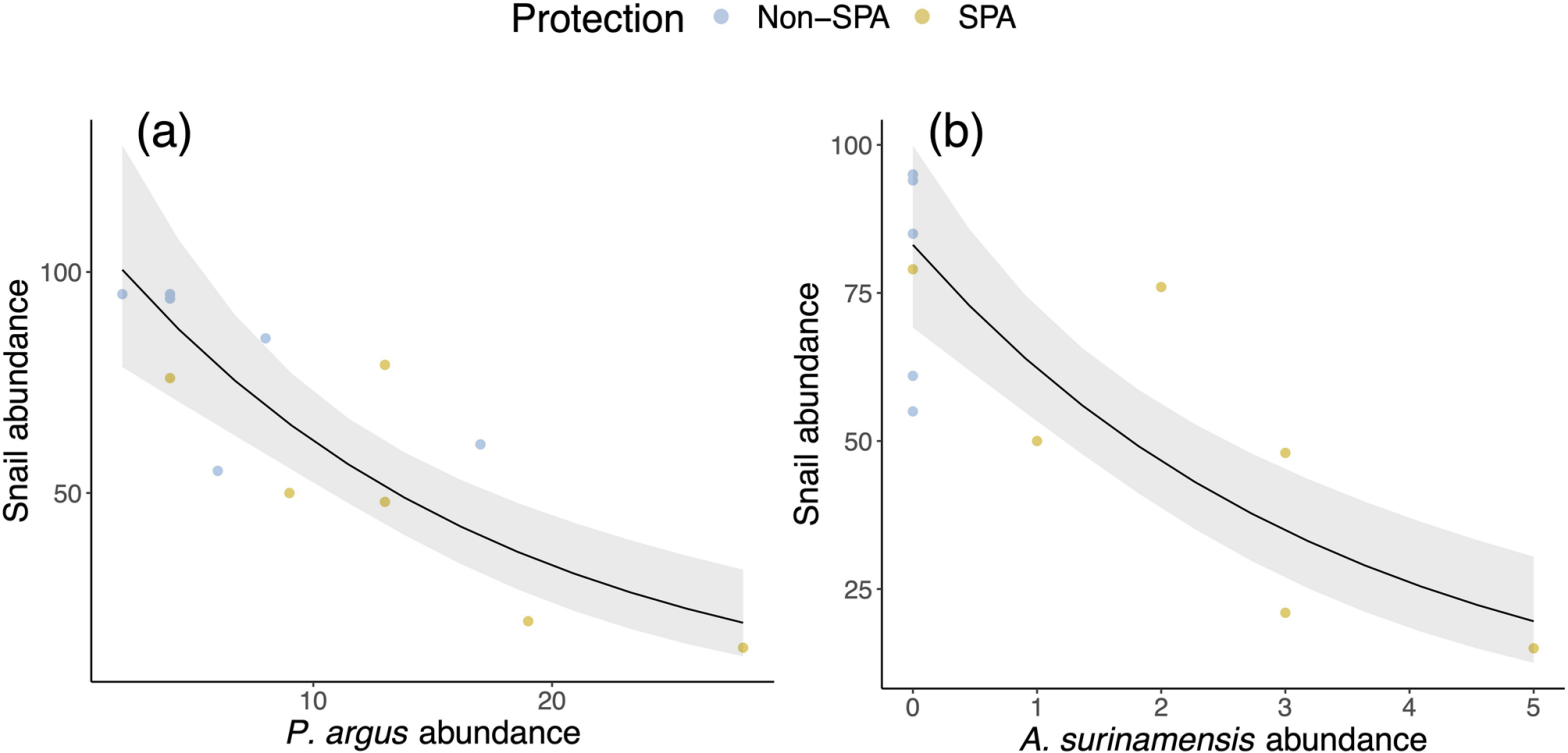
Relationship between predator abundance and *C. abbreviata* abundance. Panels show the relationship between the abundance of (**a**) spiny lobster (*P. argus*) and (**b**) black margate (*A. surinamensis*) on *C. abbreviata* abundance across all survey sites.

Looking across all surveyed potential predators, only black margate (*A. surinamensis*) and Caribbean spiny lobster (*Panulirus argus*) abundance were related to *C. abbreviata*. In both cases, we found strong, negative correlations with *C. abbreviata* abundance (*P. argus*: z_10_ = − 5.07, *P* < 0.0001, Fig. 5A; *A. surinamensis*: z_10_ = − 5.50, *P* < 0.0001, Fig. 5B), where an increase of one black margate was associated with a 1.3-fold decrease in snails per m^2^ and an increase in one spiny lobster was associated with a 1.1-fold decrease in snails per m^2^. No significant relationships were found between snails and the other individual potential predator species surveyed (see Supplementary Table S2 online).

## Discussion

In recent years, the overabundance of corallivores has become a major threat to coral reef systems around the world^2,8,32^. In the Caribbean, the common coral-eating snail *Coralliophila abbreviata* is viewed as a significant roadblock to regional efforts to recover and restore declining coral populations^17,25,28^. Despite this, the factors regulating local *C. abbreviata* abundances are not well understood, including the relative importance of available coral prey food resources and the abundance—and even identity—of their predators^30,34^. Here, we surveyed six protected and six unprotected coral reef sites spanning the Florida Keys reef tract to examine relationships between bottom-up and top-down forces on the local abundance of *C. abbreviata* in the context of marine site protection.

Across all sites surveyed, we found that snail abundance increased with increasing coral cover. However, comparing protected vs. unprotected areas, we found snail abundance was significantly lower in protected areas, despite the fact that coral cover was generally higher, suggesting an important role of site protection on *C. abbreviata* abundance. Although predator abundance did not change with SPA-designation, protected areas did have significantly higher predator richness, indicating that some key predators may be present or more abundant in protected areas and that these critical predators may be reducing snail abundance inside of protected areas, even where coral cover is generally higher. Lower snail abundance was strongly correlated with higher abundances of two predator species—black margate (*Anisotremus surinamensis*; Family Haemulidae) and Caribbean spiny lobster (*Panulirus argus*), which may be important *C. abbreviata* predators and should be studied more closely in the field. These results suggest that protected areas may influence local *C. abbreviata* populations by protecting key predator species and promoting top-down control of snails – a trophic cascade that appears more important than the increased food availability inside of protected areas. However, this may also be partially due to the relatively small difference between coral cover inside verses outside of SPAs in the Florida Keys.

Numerous factors regulate populations, including food resources, predation pressure, and recruitment. If *C. abbreviata* abundances are limited by their coral food resources, areas with higher cover or abundance of coral prey should correspond to higher abundances of *C. abbreviata*. Across all of our survey sites, we found relatively low coral cover across the sites in our study (ranging from 0.6 to 4.9% coral cover^49^) and increasing snail abundances correlated with increases with local coral cover. This suggests that food resources could be an important factor regulating snail abundances and increases in coral cover are likely to lead to increases in snail density, something that has been observed in restoration sites^32^. Our coral cover estimates are similar to other surveys in the Florida Keys (e.g. ~ 6% in 2010). However, coral cover at our survey sites may have been particularly low in 2015 due to a severe coral bleaching event in Florida in 2014, and results of our surveys may have been different with greater differences in coral cover between protected and non-protected areas. Further, we recognize that with only twelve sites these values may not be representative of the entire Florida Keys, but they do provide an important start for assessing the role of corallivores in Caribbean systems.

Our results revealed that marine protection was one of the most important factors governing snail abundance. For instance, we found that a SPA designation was associated with a 3-fold decrease in snail abundance. Given that SPAs generally had more coral cover (SPAs: 2.5 ± 1.7% cover; non-SPAs: 0.9 ± 0.3% cover), but fewer snails, coral cover does not explain this relationship. Another mechanism that could be operating is that differences in snail abundance in and out of SPAs are driven by top-down effects. In our study, for instance, black margate (*A. surinamensis*) and the Caribbean spiny lobster (*P. argus*) were generally more abundant in protected areas and had strong inverse associations with *C. abbreviata* abundance. Indeed, other studies have documented the positive effects of no-take zones on abundances of black margate^50^ and Caribbean spiny lobster^51,52^, while previous research in Florida has found that SPA designation positively affects overall fish biomass^38^. Thus, these findings suggest that higher abundances of gastropod predators may be important for reducing local populations of *C. abbreviata* in protected areas.

Our results suggest that in areas where reef protection does not dramatically alter coral cover but does affect predator populations, reef protection may benefit corals by reducing *C. abbreviata* abundances. Both observational and experimental studies in other regions have found similar relationships, where reduced fishing pressure or site protection is associated with reduced densities of corallivorous invertebrates like sea stars^20,21^ and gastropods^15,19^. For instance, a study in Fiji found 5–35 times higher densities of the coral-eating snail *Coralliophila violacea* in unprotected areas relative to neighboring MPAs, potentially due to fewer predatory fish in unprotected sites^33^. However, marine protected area designs often tend to focus on protecting key herbivores and facilitating coral health through reduced macroalgal cover^4,5,7,53^. The results of our study build on a growing body of research showing the importance of marine protection in conserving other key trophic interactions and ecological processes that are important for coral populations and reefs. Densities of *C. abbreviata* similar or higher to those found in this study have been reported in other locations in the Caribbean (e.g., Jamaica^16^: 0.2–0.6 snails per m^2^; Barbados^54^: 13 snails per m^2^) and with other corallivorous gastropods such as *Drupella* spp. in Australia^9,16,55^. Additional research is needed to determine whether these results are consistent across geographies with *C. abbreviata* and with coral-eating snails in diverse regions.

Since predators of *C. abbreviata* remain largely unknown or unconfirmed in the literature, we also sought to identify potential predators of this snail. We found black margate (*A. surinamensis*) had the strongest relationship with *C. abbreviata*. Although no literature exists for the relationship between *Coralliophila* spp. and black margate (or for other members of the family Haemulidae), haemulids are general specialists on mollusks, with diverse gastropods making up a large portion of their diets in previous studies^42^. Previous research from the 1960s using gut content analysis found the long-spined urchin *Diadema antillarum* dominated the diet of black margate^42^. Dramatic declines in *D. antillarum* populations across the Caribbean and their continued ecological extinction from reefs in the Florida Keys may have required black margate to switch to other, more abundant invertebrates. Smaller black margate have also been reported to rely more heavily on gastropods in their diet. Additionally, black margates are one of the largest fish in the Haemulidae family (averaging 45 cm long^35^), potentially allowing them to prey on thicker-shelled snails like *C. abbreviata*. The Caribbean spiny lobster was also inversely correlated with *C. abbreviata* abundance. While no studies to date confirm this predator–prey relationship under natural settings, this species is the most commonly suspected predator of *C. abbreviata* snails^30,35,41^ and one previous study and personal observation (author, E. Shaver) has observed lobsters feeding on *C. abbreviata* in the laboratory^30^.

Due to limitations in our study design, we were unable to survey potentially important nocturnal predators, such as the spotted spiny lobster (*Panulirus guttatus*) or the common octopus (*Octopus vulgaris*). Additionally, the deltoid rock snail (*Thais deltoidea*) has been confirmed as a predator *C. abbreviata* but was not surveyed as it had yet to be identified as a predator at the time of this study^34^. Further investigations should seek to examine how predation by the deltoid rock snail varies across protected and unprotected sites in Florida, examine relationships with additional nocturnal or cryptic species, or following this study, further quantify the magnitude of population reduction of *C. abbreviata* by its predators. While further investigation is needed, the research presented here establishes a baseline for future studies examining top-down control on corallivores in Florida and the Caribbean.

In addition to differences in abundance, *C. abbreviata* snails were on average 2 mm longer in protected sites. One hypothesis for this difference in snail size considers that the more abundant and diverse predator assemblages in protected areas may more successfully feed on smaller snails, affecting the size structure of *C. abbreviata*. Black margate are gape-limited predators that are restricted to feeding on prey smaller than their mouths. Likewise, spiny lobsters use their mandibles to shred the outer lip of *C. abbreviata* shells in the absence of large claws (E. Shaver, *personal observation*), which is more difficult with larger snails that have thicker shells. One previous study has also suggested this relationship between Caribbean spiny lobsters and *C. abbreviata* size, hypothesizing that heavy harvesting of lobsters in Florida has reduced average lobster size and subsequently their ability to successfully prey on large *C. abbreviata*^41^. Similarly, other research has found that kelp forests are converted to barrens by urchin overgrazing when the average size of spiny rock lobsters (*Jasus edwardsii*) decreases outside of protected areas due to incompatibility between predator and prey sizes^56^. Another explanation for the difference in snail size between protected and unprotected sites could be that different abundances of specific coral species result in differently sized *C. abbreviata*, as other studies in Florida have found that *C. abbreviata* growth rates and life span (which contribute to average size) vary depending on their coral species prey^40^. Although this specific relationship was not examined in this study, we did not find an effect of coral cover on snail size and coral cover was generally similar across sites.

Corallivory harms living corals through direct tissue mortality and sublethal effects on coral fitness. Our surveys showed that a common corallivore, the short coral snail *Coralliophia abbreviata*, is abundant on reefs throughout Florida as it in on many reefs in the Caribbean^9^. The results of our study suggest that while local abundances of *C. abbreviata* are related to the amount of living coral food resources, their abundances also appear to be regulated within protected areas, potentially reducing chronic stress on corals that leads to mortality^16,28,30^, disease^26,27,29^, and reduced growth^28^, fecundity^40^, and hampered coral resilience^9–11^. This relationship may be due to the presence or increased abundances of key *C. abbreviata* predators within protected sites. With future research on these relationships, specific regulations aimed at increasing local gastropod predator populations may help reduce the impacts of *C. abbreviata* on recovering coral reefs, reducing the need for human interventions, such as manual snail removal efforts, that can be cost and labor intensive.

## Supplementary information

Supplementary Information.

## Data Availability

The datasets generated and analyzed for this study are available in the Dryad repository at https://doi.org/10.5061/dryad.mpg4f4qwf.
