# Supplementary material for: Relationships between a common Caribbean corallivorous snail and protected area status, coral cover, and predator abundance
Source: Sci Rep. 2020 Oct 5;10:16463. doi: 10.1038/s41598-020-73568-1 (PMC7536437; doi:10.1038/s41598-020-73568-1)
Supplement: Supplementary file 1 — Supplementary Information. [file 41598_2020_73568_MOESM1_ESM.pdf]

Supplementary Information for

Relationships between a common Caribbean corallivorous snail and protected area status, coral cover, and predator abundance

Elizabeth C. Shaver[1,2*], Julianna J. Renzi[2], Maite G. Bucher[3], Brian R. Silliman[2]

[1] The Nature Conservancy, Arlington, VA, USA

[2] Division of Marine Science and Conservation, Nicholas School of the Environment, Duke University, Beaufort, NC, USA

[3] Department of Environmental Health Science, College of Public Health, University of Georgia, Athens, GA, USA

This PDF file includes:

Supplementary Table S1. Densities of *Coralliophila abbreviata* (number per m^2) and coral cover (%, mean ± SD) for all sites surveyed.

Supplementary Table S2. Results from negative binomial general linear models of *Coralliophila abbreviata* abundance versus potential snail predators. Bolded values are significant after the Bonferroni correction.

Supplementary Table S1. Densities of *Coralliophila abbreviata* (number per m^2) and coral cover (%, mean ± SD) for all sites surveyed.

Reef Site	Protection Status	*C. abbreviata* density	Coral Cover
Conch Reef	Fishing allowed	0.26	0.93 ± 0.93
Horseshoe Reef	Fishing allowed	0.16	0.66 ± 0.46
American Reef	Fishing allowed	0.16	0.92 ± 0.73
Pickles Reef	Fishing allowed	0.16	1.45 ± 1.38
Crocker Reef	Fishing allowed	0.09	0.60 ± 0.50
Delta Shoals	Fishing allowed	0.10	0.98 ± 0.63
Molasses Reef	SPA / No-take	0.10	1.96 ± 1.16
Western Sambo	SPA / No-take	0.15	4.07 ± 2.53
Carysfort Reef	SPA / No-take	0.08	2.16 ± 1.20
Conch Reef SPA	SPA / No-take	0.04	0.88 ± 0.58
Looe Key	SPA / No-take	0.13	4.93 ± 4.50
Alligator Reef	SPA / No-take	0.03	0.77 ± 0.52

Supplementary Table S2. Results from negative binomial general linear models of *Coralliophila abbreviata* abundance versus potential snail predators. Bolded values are significant after the Bonferroni correction.

**Common Name**	**Species**	**Coefficient (SE)**	**df**	**P-value**
Black Margate	*Anisotremus surinamensis*	-0.061 (0.012)	11	**< 0.0001**
Porkfish	*Anisotremus virginicus*	0.017 (0.027)	11	0.518
Spanish Hogfish	*Bodianus rufus*	-0.021 (0.023)	11	0.359
Porcupinefish	*Diodon hystrix*			
Slippery Dick	*Halichoeres bivittatus*	0.002 (0.004)	11	0.529
Caesar Grunt	*Haemulon carbonarium*	-0.001 (0.004)	11	0.891
Spanish Grunt	*Haemulon macrostomum*	-0.058 (0.032)	11	0.069
Sailor's Choice	*Haemulon parra*	-0.074 (0.127)	22	0.560
White Grunt	*Haemulon plumineri*	-0.001 (0.002)	11	0.368
Puddingwife	*Halichoeres radiatus*	-0.030 (0.020)	11	
Bluestriped Grunt	*Haemulon sciurus*	0.0001 (0.001)	11	0.848
Hogfish	*Lachnolaimus maximus*	-0.019 (0.061)	11	0.758
Spiny Lobster	*Panulirus argus*	-0.289 (0.052)	11	**< 0.0001**
